# Insights of fibroblast growth factor receptor 3 aberrations in pan-cancer and their roles in potential clinical treatment

**DOI:** 10.18632/aging.203175

**Published:** 2021-06-23

**Authors:** Juanni Li, Kuan Hu, Jinzhou Huang, Lei Zhou, Yuanliang Yan, Zhijie Xu

**Affiliations:** 1 Department of Pathology, Xiangya Hospital, Central South University, Changsha 410008, Hunan, China; 2 Department of Hepatobiliary Surgery, Xiangya Hospital, Central South University, Changsha 410008, Hunan, China; 3 Department of Oncology, Mayo Clinic, Rochester, MN 55905, USA; 4 Department of Anesthesiology, Third Xiangya Hospital of Central South University, Changsha 410008, Hunan, China; 5 Department of Pharmacy, Xiangya Hospital, Central South University, Changsha 410008, Hunan, China; 6 National Clinical Research Center for Geriatric Disorders, Xiangya Hospital, Central South University, Changsha 410008, Hunan, China

**Keywords:** FGFR3, alteration, fusion, prognosis, pan-cancer

## Abstract

Fibroblast growth factor receptor 3 (FGFR3) alters frequently across various cancer types and is a common therapeutic target in bladder urothelial carcinoma (BLCA) with FGFR3 variants. Although emerging evidence supports the role of FGFR3 in individual cancer types, no pan-cancer analysis is available. In this work, we used the open comprehensive datasets, covering a total of 10,953 patients with 10,967 samples across 32 TCGA cancer types, to identify the full alteration spectrum of FGFR3. FGFR3 abnormal expression, methylation patterns, alteration frequency, mutation location distribution, functional impact, and prognostic implications differed greatly from cancer to cancer. The overall alteration frequency of FGFR3 was relatively low in all cancers. Targetable mutations were mainly detected in BLCA, and S249C, Y373C, G370C, and R248C were hotspot mutations that could be targeted by an FDA approved erdafitinib. Genetic fusions were mainly observed in glioma, followed by BLCA. FGFR3-TACC3 was the most common fusion type which was proposed as novel therapeutic targets in glioma and was targetable with erdafitinib in BLCA. Lung adenocarcinoma (LUAD) and lung squamous cell carcinoma (LUSC) were two lung cancer subtypes, FGFR3 fusion and hotspot mutation like S249C were observed more commonly in LUSC but not in LUAD. DNA methylation was correlated with the expression of FGFR3 and its downstream genes in some tumors. FGFG3 abnormal expression and alterations exhibited clinical correlations with patient prognosis in several tumors. This work exhibited the full alteration spectrum of FGFR3 and indicated several new clues for their application as potential therapeutic targets and prognostic indicators.

## INTRODUCTION

The family of fibroblast growth factor receptor (FGFR) comprises four receptor tyrosine kinases (FGFR1, FGFR2, FGFR3, and FGFR4) involved in several critical cellular processes, such as angiogenesis, proliferation, differentiation, and metabolism [1–5]. *FGFR3* is highly expressed in osteoblasts and chondrocytes, and has classically been known to play critical roles in osteogenesis, development, and bone maintenance [6, 7]. Additionally, FGFR3 signaling has been reported to overlap with several known oncogenic pathways such as RAS/EGFR/ERK/PI3K/AKT pathway and has been implicated in epithelial-mesenchymal transition (EMT) in some tumors [8, 9].

In recent years, along with improvements in clinical genetic testing techniques in oncology, more *FGFR3* gene alterations are discovered and implicated in a wide range of cancers [10–12]. The most common *FGFR3* mutation type detected in tumors is S249C, and the mutagenic mechanism is mediated by catalyzing polypeptide-like (APOBEC) through an apoprotein B mRNA editing enzyme [13]. More recently, erdafitinib, a pan-FGFR targeted inhibitor, was approved by the FDA in April 2019 for advanced urothelial carcinoma with FGFR3 hotspot mutation like S249C as the first molecularly targeted therapy [14–16]. However, it should be noted that not all FGFR3 mutations confer sensitivity to FGFR inhibitors. For example, V555M mutation which is detected in multiple myeloma confers resistance to FGFR3 inhibitors through the steric hindrance of the kinase-inhibitor interaction [17]. Moreover, recently, gene fusions involving FGFR3 have been observed in some cancer types, and glioma harbors the highest FGFR3 fusion rate. FGFR3 -TACC3 is the most identified fusion type, followed by fusions containing BICC1, TACC2, and NPM1 [18, 19]. This fusion type was reported to be able to confer constitutive kinase activity of FGFR3 and promotion of cell transformation and proliferation [20, 21]. These FGFR3 alterations are identified as the oncogenic drivers and are also considered to be potential therapeutic targets. Nowadays, multiple FGFR inhibitors are in the pipeline, further FDA approval is possible, and it is highly likely their application in targeted treatment will extend to other tumor types.

As previous studies on FGFR3 genetic alterations in cancer are limited to the individual cancer types and/or to the insufficient sample sizes, a comprehensive analysis and view across various tumor types of TCGA to investigate their significance have not been explored. In this work, we analyzed the large datasets from TCGA and fill this vacancy in a comprehensive way. We first systematically profiled FGFR3 expression, methylation, genetic alterations, and their clinical and therapeutic implications across 32 TCGA cancer types covering 10,967 tumor samples. Additionally, the survival association between FGFR3 aberration patterns and prognosis in distinct cancer types was conducted to explore its potential therapeutic implication. Conclusively, our analysis results highlight the important role of FGFR3 in tumorigenesis and provide potential and promising therapeutic targets across different cancers.

## RESULTS

### Expression and methylation level of FGFR3 in different cancer types

**Figure 1:**
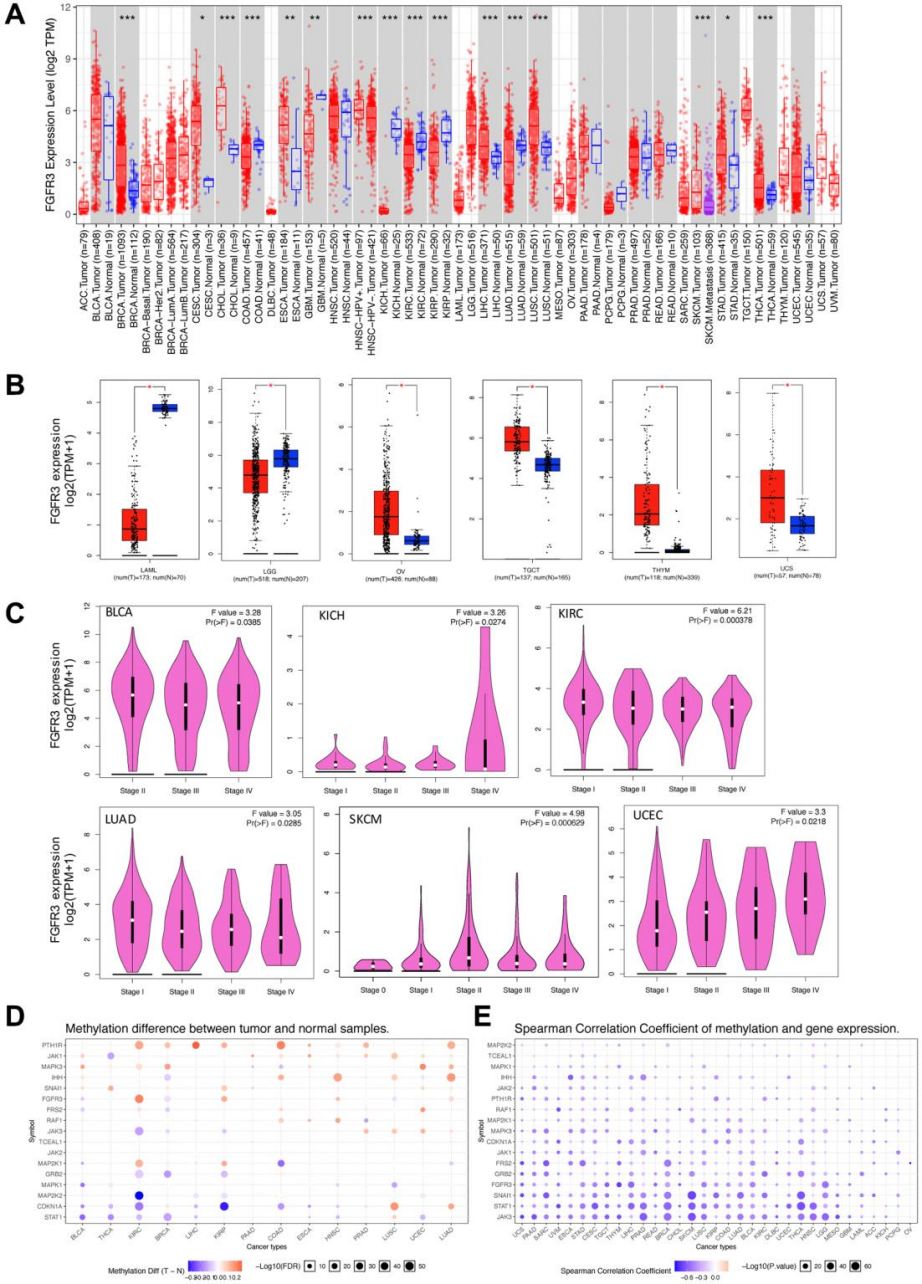
**Fibroblast growth factor receptor 3 (FGFR3) mRNA expression and DNA methylation in The Cancer Genome Atlas (TCGA) tumor tissues.** (**A**) The mRNA expression of FGFR3 in different cancers or specific cancer subtypes from TIMER2. The log2 [TPM (Transcripts per million)] was applied for log-scale. (**B**) For LAML, LGG, OV, TGCT, THYM, and UCS from the TCGA project, their corresponding normal tissues of the GTEx database were included as controls based on the GEPIA2 portal. The log2 (TPM +1) was applied for log-scale. (**C**) FGFR3 mRNA expression levels were analyzed by the main pathological stages of BLCA, KICH, KIRC, LUAD, SKCM, and UCEC. The log2 (TPM +1) was applied for log-scale. (**D**) Bubble map showing the differential methylation of FGFR3 and its downstream genes between tumors and matched normal samples. Blue dots, downregulated methylation in tumors. Red dots, upregulated methylation in tumors. (**E**) Bubble map depicting the relative methylation and expression differences of FGFR3 and its downstream genes between tumors and matched normal samples by size and color, respectively. Blue dots, upregulation in methylation level but downregulation in expression level. Red dots, upregulation in both methylation and expression levels of indicated genes. ^*^P < 0.05; ^**^P < 0.01; ^***^P < 0.001.

FGFR3 abnormal expression has been observed in various cancer types [22, 23]. In this work, we provided a more comprehensive analysis of FGFR3 expression. First of all, we explored the expression pattern of FGFR3 among different types of normal tissues by the GTEx portal. FGFR3 expression exhibited a broad spectrum across different tissues. FGFR3 showed the highest expression in the skin and almost no expression in the EBV-transformed lymphocytes (Supplementary Figure 1). Next, FGFR3 expression was compared across 32 TCGA cancer types (Supplementary Table 1). As shown in Supplementary Figure 2A, FGFR3 expression across different cancers was dramatically different, indicating that high FGFR3-expressing cancers may have some genetic features that lead to the increased FGFR3 expression. According to the interquartile range, FGFR3 expression spread varied in several cancers more than others, for example, skin cutaneous melanoma (SKCM) had a wide spread while testicular germ cell tumors (TGCT) had a narrow spread, which may be on account of some cancer types including several subtypes and thus having more genetic diversity (Supplementary Figure 2A). Moreover, we evaluated the expression difference of FGFR3 between tumors and the corresponding normal tissues profiled in TCGA. As shown in Figure 1A, significantly differential expression of FGFR3 was observed in 16 tumor types, with 10 tumor types upregulated [breast invasive carcinoma (BRCA), cervical squamous cell carcinoma and endocervical adenocarcinoma (CESC), cholangiocarcinoma (CHOL), esophageal carcinoma (ESCA), head and neck squamous cell carcinoma (HNSC-HPV), LICH, LUSC, SKCM, stomach adenocarcinoma (STAD), thyroid carcinoma (THCA)] and 6 tumor types downregulated [colon adenocarcinoma (COAD), GBM, kidney chromophobe (KICH), kidney renal clear cell carcinoma (KIRC), kidney renal papillary cell carcinoma (KIRP), LUAD]. After adding GTEx normal tissues as control, we further compared FGFR3 expression difference between the normal tissues and tumors of adrenocortical carcinoma (ACC), lymphoid neoplasm diffuse large B-cell lymphoma (DLBC), acute myeloid leukemia (LAML), LGG, ovarian serous cystadenocarcinoma (OV), sarcoma (SARC), TGCT, thymoma (THYM), and uterine carcinosarcoma (UCS). Upregulated expression of FGFR3 was observed in 4 cancer types (OV, TGCT, THYM, and UCS) and downregulated expression of FGFR3 was observed in 2 cancer types (LAML and LGG) (Figure 1B, Supplementary Figure 3A). Furthermore, we explored the correlation between FGFR3 expression and the tumor pathological stages by the GEPIA2 approach. It was found that FGFR3 expression was correlated with tumor pathological stages in several cancer types, including BLCA, KICH, KIRC, LUAD, SKCM, and uterine corpus endometrial carcinoma (UCEC) (Figure 1C, all *P* < 0.05). However, no correlation was found in the remaining cancer types (Supplementary Figure 3B–3F, all *P* > 0.05).

DNA methylation was reported to be strongly associated with the change of gene expression in tumors [24, 25]. Therefore, we applied the GSCALite approach to explore the methylation status of FGFR3 and its downstream genes in various cancer types of TCGA. As shown in Figure 1D, up-regulated methylation of FGFR3 was observed in KIRC, KIRP, HNSC, UCEC, and LUAD, while down-regulated methylation of FGFR3 was found in BLCA, BRCA, and LUSC. In addition, the expression of FGFR3 and its downstream genes were found to be most negatively correlated with FGFR3 methylation status, with only a few positive correlations (Figure 1D).

### FGFR3 somatic mutation patterns in different cancer types

**Figure 2:**
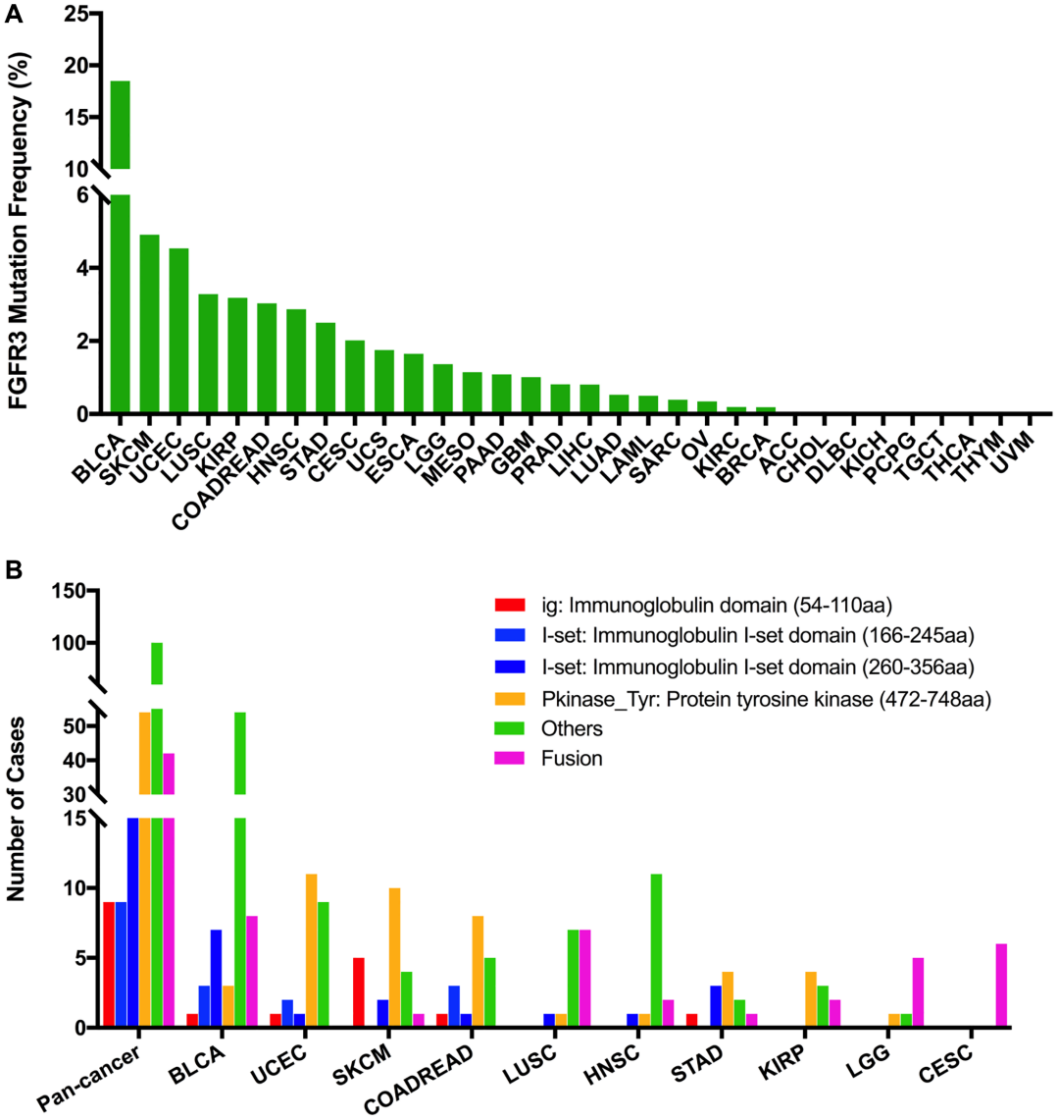
**FGFR3 mutation distribution in different cancer types of TCGA and protein functional domains.** (**A**) The mutation frequency of FGFR3 across various tumor types. (**B**) FGFR3 mutation distribution in different protein functional domains in all and top ten tumor types. Abbreviation: aa: amino acid.

The total mutation frequency of FGFR3 was 2.13% for all cancer samples (234/10,967) across various cancer types of TCGA. The tumor sample number from different cancer types varied from 36 (CHOL) to 1,084 (BRCA) (Supplementary Table 2). Those cancer types with too few samples such as CHOL might not represent the full landscape of FGFR3 mutation status. Moreover, as shown in Figure 2A, BLCA (18.5%), SKCM (4.9%), and UCEC (4.5%) were the most common cancer types with FGFR3 mutations. On the contrary, almost no FGFR3 mutations were observed in ACC, CHOL, DLBC, KICH, pheochromocytoma and paraganglioma (PCPG), TGCT, THCA, THYM, and uveal Melanoma (UVM).

**Figure 3:**
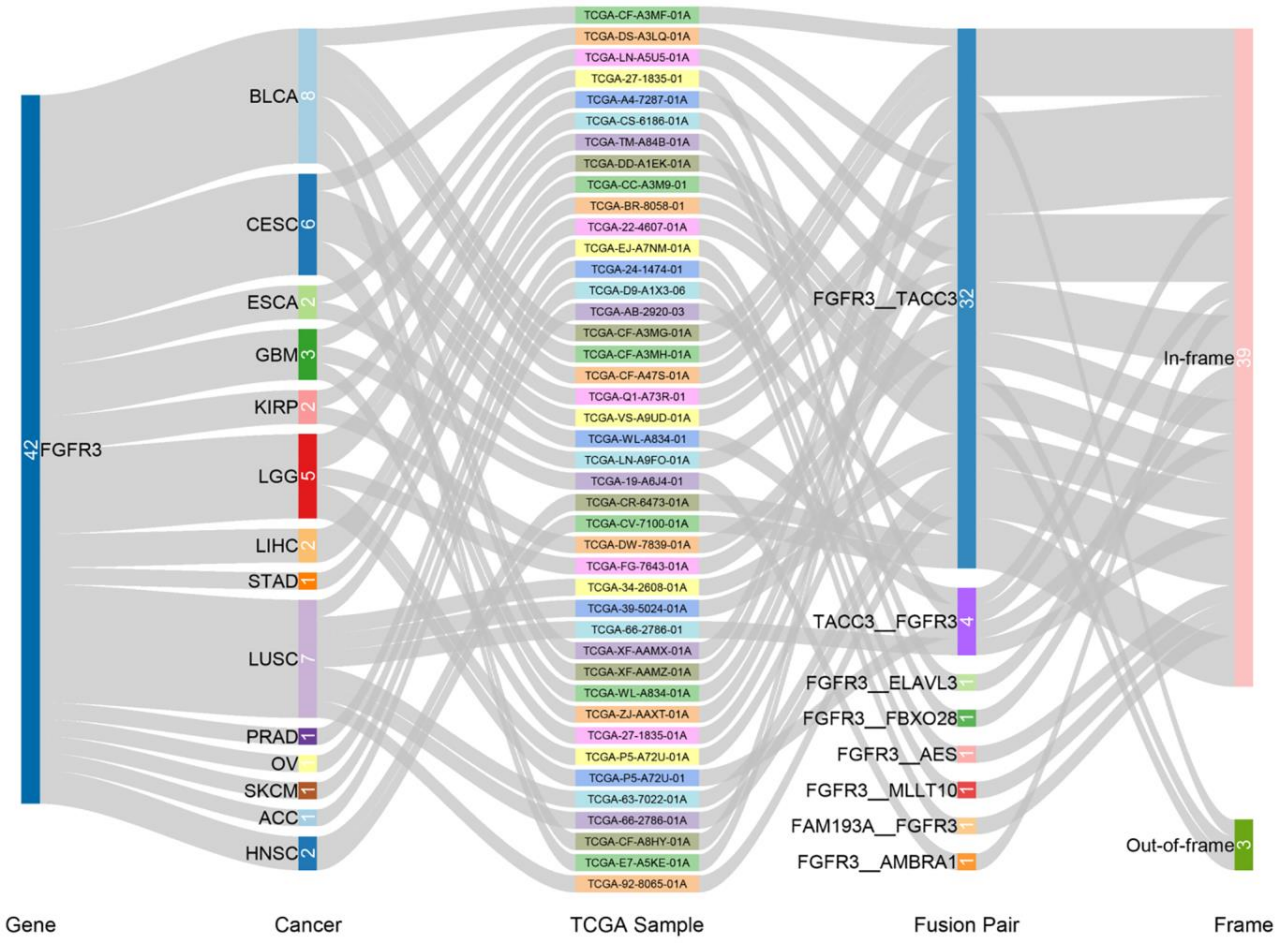
Fusion gene of FGFR3 across 32 TCGA tumor types.

We observed 234 FGFR3 somatic mutations across 32 TCGA cancers, among these mutations, 42 FGFR3 mutations belonged to fusion. As shown in Figure 3, fusion transcripts of FGFR3 were observed in BLCA (8), LUSC (7), CESC (6), LGG (5), GBM (3), ESCA (2), KIRP (2), liver hepatocellular carcinoma (LIHC) (2), HNSC (2), STAD (1), prostate adenocarcinoma (PRAD) (1), OV (1), SKCM (1), ACC (1). The highest number of fusion transcripts was found in BLCA (eight FGFR3_TACC3), followed by LUSC (six FGFR3_TACC3, one TACC3_FGFR3), CESC (five FGFR3_TACC3, one TACC3_FGFR3), and LGG (two FGFR3_TACC3, one TACC3_FGFR3, one FGFR3_ELAVL3, one FGFR3_FBXO28). FGFR3_TACC3 was the most common fusion transcripts of FGFR3 (32/42) and distributed in different cancer types [BLCA (8), LUSC (6), CESC (5), ESCA (2), LGG (2), HNSC (2), KIRP (2), LIHC (2), GBM (1), STAD (1), ACC (1)]. TACC3 is a tumor-associated protein that has been found to play critical roles in the development of various cancer types, such as ovarian cancer, hepatocellular carcinoma, glioblastoma, and so on. It was also involved in several crucial cellular events, like cell differentiation, growth, transcriptional regulation, and the regulation of centrosome and microtubule [26]. Most fusion transcripts of FGFR3 were classified as the in-frame, while three FGFR3_TACC3 (one BLCA, one LUSC, one LIHC) were classified as the out-of-frame.

FGFR3 was found to have four functional domains based on the Pfam database, containing the PKinase_Tyr domain (472–748 aa), I-set domain (260–356 aa), I-set domain (166–245 aa), and ig domain (54–110 aa). In this analysis, 234 FGFR3 mutations were detected in various cancer types of TCGA and distributed across different FGFR3 functional domains. As shown in Figure 2B, the other domain whose function was barely known was the most frequently mutated domain of FGFR3 (100 samples), followed by the Pkinase_Tyr domain (54 samples), the I-set (260–356 aa, 20 samples), the I-set (166–245 aa, 9 samples), and the ig domain (9 samples). Moreover, the location distribution of FGFR3 mutations differed greatly across different TCGA cancers. Mutations in UCEC, SKCM, colon adenocarcinoma/rectum adenocarcinoma (COADREAD), STAD, and KIRP were most commonly distributed in the Pkinase_Tyr domain. Mutations in BLCA and HNSC were primarily located in the other domain and amounted to around two-thirds of all FGFR3 mutations. Mutations in LGG and CESC were mainly fusion, especially for CESC, fusion was the only mutation type in this cancer type. In LUSC, fusion and mutations in the other domain were equally common (Figure 2B and Supplementary Table 3).

The 234 FGFR3 mutations mentioned above were classified into three categories based on mutation functional impact on FGFR3 protein coding, including missense mutations (177 samples), fusion (42 samples), and truncating mutations (15 samples). The most common mutation positions of FGFR3 were S249C (41 samples) and Y373C (11 samples), both of which were located in the other domain. Mutations at S249C were most observed in BLCA samples which were amounted to nearly three-quarters of all mutations in this position (32/41). The S249C was an FGFR3 hotspot mutation and known to be oncogenic. Patients with metastatic urothelial tumor carrying S249C mutation could be treated with the pan-FGFR targeted inhibitor, erdafitinib, which was approved by Food and Drug Administration (FDA) [27, 28]. The other cancer types carrying S249C mutation were HNSC (4 samples), LUSC (4 samples), and KIRP (one sample). However, different from that in BLCA, the clinical utility of targeted drugs in these three cancer types with S249C mutation was still unknown. Similarly, BLCA harbored the largest proportion of mutations at Y373C (8/11), followed by KIRP (two samples) and UCEC (one sample). BLCA with a mutation at this position could be also treated with the FDA-approved erdafitinib (Supplementary Figure 4A) [27, 28]. Moreover, as shown in Figure 2A and Supplementary Figure 4B, BLCA had the highest frequency of FGFR3 mutational alterations. S249C was most common among BLCA mutation samples (32 samples), followed by Y373C (8 samples), G370C (5 samples), and R248C (3 samples). All these four mutation positions of FGFR3 were oncogenic and were FDA recognized biomarker predictive of response to an FDA-approved drug, such as erdafitinib [27, 28]. For mutations at K650E (two samples), S371C (two samples), and G380R (two samples) in BLCA, there was promising clinical evidence that supported these mutation positions as being predictive of response to pan-FGFR-targeted inhibitors such as Debio1347, BGJ398, AZD4547, and erdafitinib [27, 29–33]. Furthermore, in UCEC, the most mutated positions were in the Pkinase_Tyr domain. Mutation at Y373H in UCEC was considered likely oncogenic, and several laboratory data suggested that tumor cells with Y373H mutation may be sensitive to some selective FGFR-targeted inhibitors (Supplementary Figure 4C) [28, 34–36].

**Figure 4:**
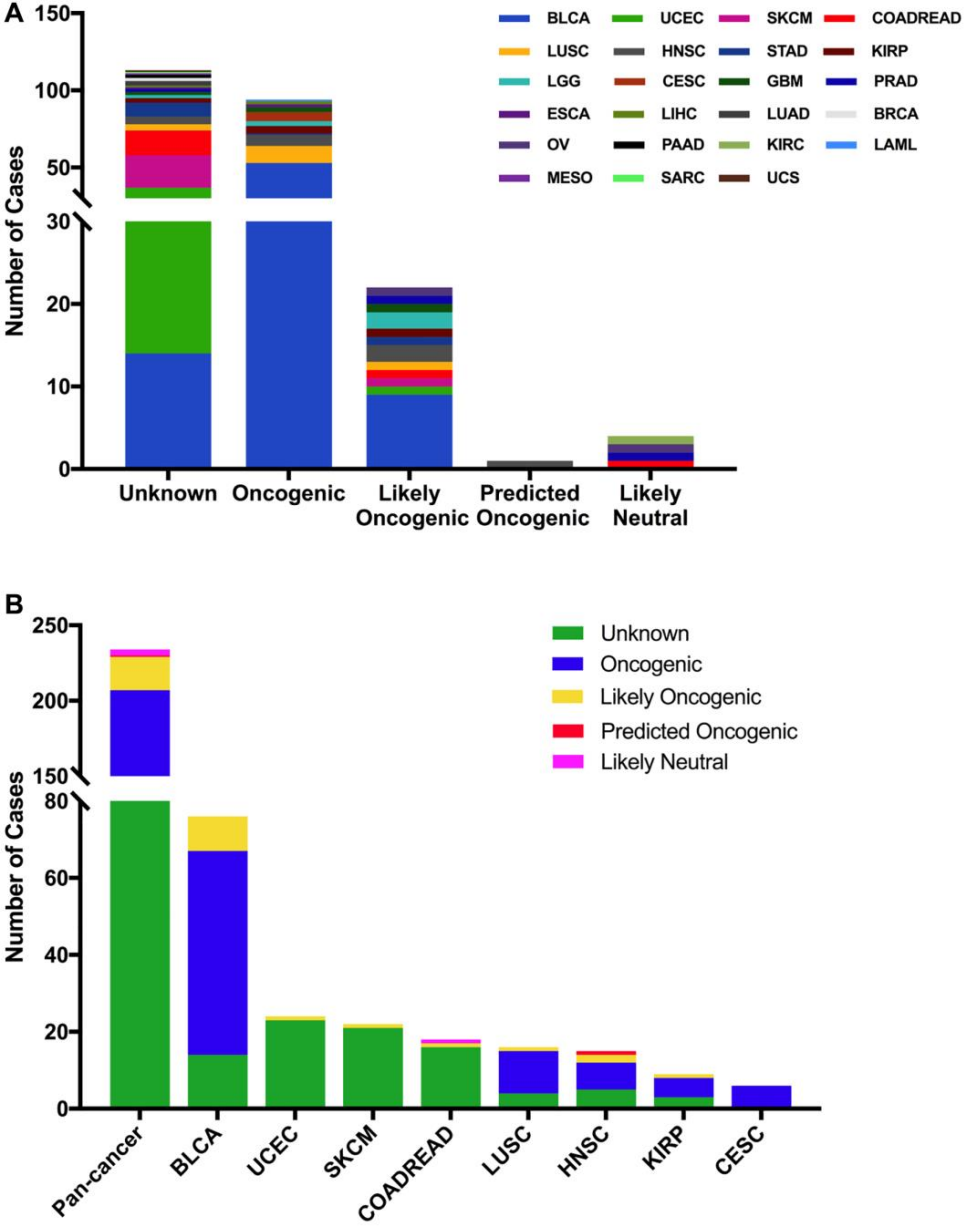
**FGFR3 mutation classification based on the functional impact on protein coding.** (**A**) FGFR3 mutations were categorized according to the functional impacts on all tumors together. (**B**) Functional impact category distribution of FGFR3 mutations in pan-cancer and the top eight tumor types.

The 234 FGFR3 mutations were divided into five classes based on their predictive significance and oncogenic effect, including unknown (113 mutations), oncogenic (94 mutations), likely oncogenic (22 mutations), predicted oncogenic (1 mutation), and likely neutral (4 mutations). As shown in Figure 4A, nearly half of FGFR3 somatic mutations were distributed in the unknown class, indicating that more research needs to be conducted to explore the role of these mutations. However, mutations that were distributed in the functional classes took up a major portion of FGFR3 mutations in several cancers such as BLCA, LUSC, HNSC, KIRP, and CESC. In BLCA and LUSC, about two-thirds of mutations belonged to the oncogenic class (53/76, 11/16, respectively), and the other mutation that was distributed in the functional class was likely oncogenic (nine mutations, one mutation, respectively). In CESC, all somatic mutations of FGFR3 belonged to oncogenic (Figure 4B).

**Figure 5:**
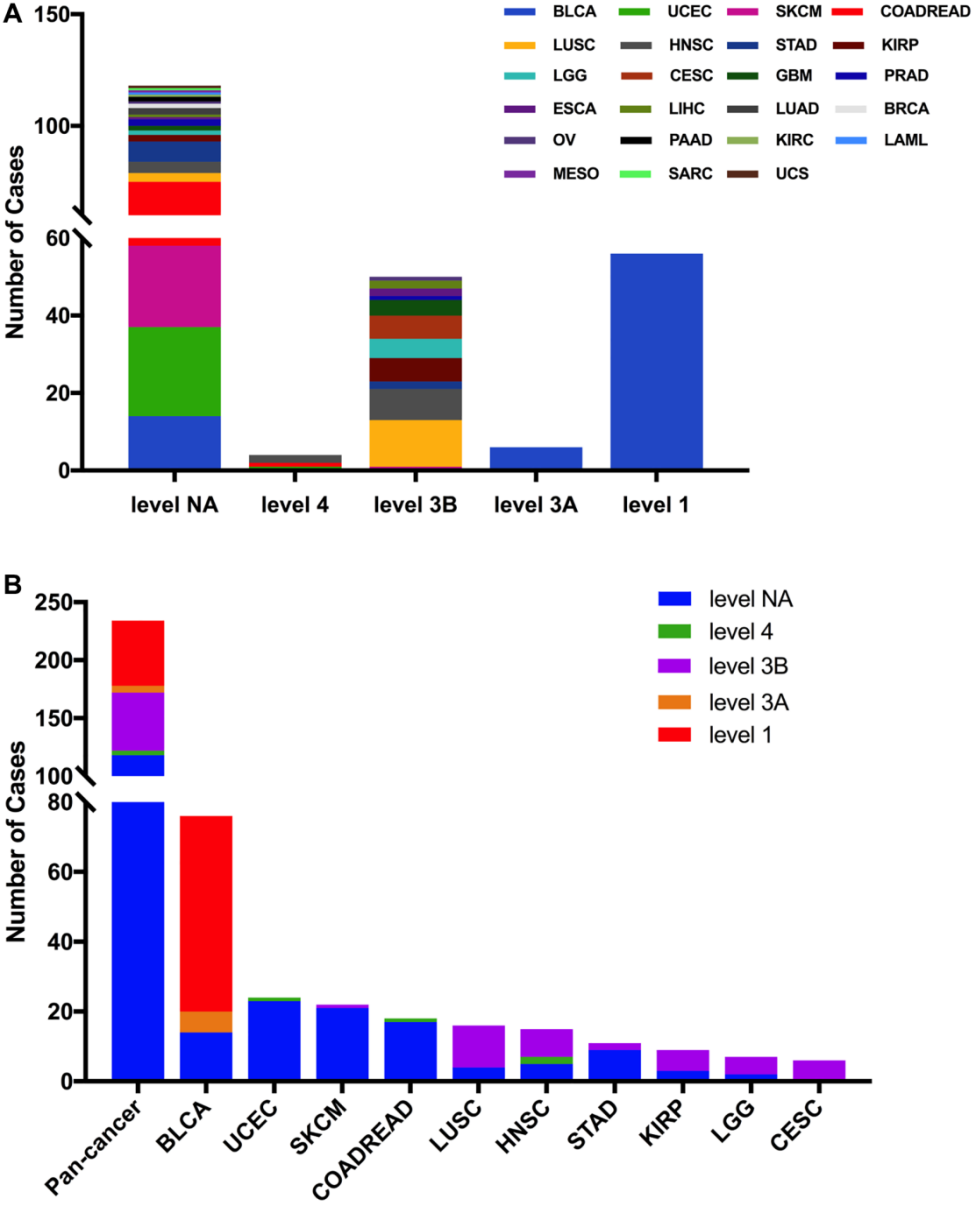
**FGFR3 mutation classification according to clinical therapeutic implications.** (**A**) FGFR3 mutations were classified according to the therapeutic implications defined by OncoKB among all tumors together. (**B**) Therapeutic implications class distribution of FGFR3 mutations in pan-cancer and the top ten tumor types.

Then we used the cBioPortal approach to analyze the clinical targeted therapy application potential of FGFR3 somatic mutations. The 234 FGFR3 mutations were classified as five levels which were defined by OncoKB [37], containing level NA (118 mutations), level 4 (four mutations), level 3B (50 mutations), level 3A (six mutations), and level 1 (56 mutations). Approximately half of the FGFR3 mutations were classified as level NA which represented no targeted therapy implication, indicating that more work was needed to improve the status of the current targeted treatment (Figure 5A). All level 1 mutations were found in BLCA, which accounted for nearly two-thirds of FGFR3 mutations. Most of these level 1 mutations were S249C, and the remaining mutations were Y373C, G370C, and R248C. BLCA patients with these level 1 mutations were suitable for targeted treatment with an FDA-approved medicine [16]. Meanwhile, there were six mutations in BLCA that belonged to level 3A, which represented that there was promising clinical evidence that supported these level 3A mutations as being predictive of response to targeted therapy. Furthermore, level 3B mutations were distributed in several cancer types such as LUSC, HNSC, KIRP, LGG, and CESC (Figure 5B).

### FGFR3 CNVs across cancer types

**Figure 6:**
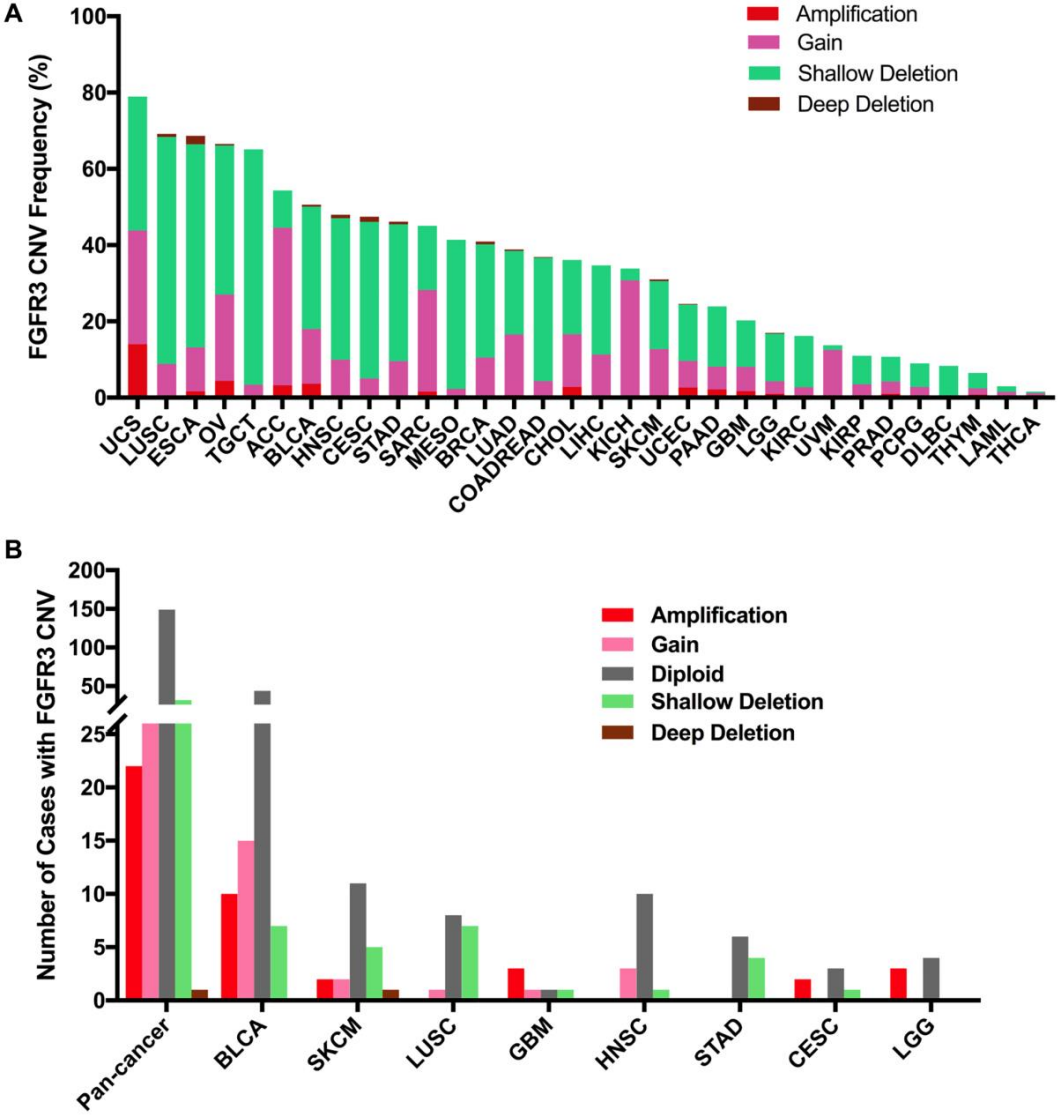
**Pan-cancer analysis of FGFR3 Copy Number Variant (CNV).** (**A**) The CNV frequency of FGFR3 across various tumor types. (**B**) FGFR3 CNV distribution in pan-cancer and the top eight tumors for the cases with FGFR3 mutations simultaneously.

Here, we explored the CNVs of FGFR3 in different cancer types. The overall CNV frequency of FGFR3 was about 34.5% (3784/10,967 samples). The most common CNV type of FGFR3 was shallow deletion (2625 samples), then gain (1000 samples), amplification (120 samples), and deep deletion (39 samples). Most of the amplification were mainly detected in UCS, OV, and BLCA, while most of the deep deletion was found in ESCA, HNSC, CESC, and BRCA (Figure 6A). The most common cancer types with FGFR3 CNVs were UCS (78.9%), LUSC (69.2%), ESCA (68.7%), OV (66.5%), and TGCT (65.1%). On the contrary, THCA (1.6%), LAML (3.0%), THYM (6.5%), DLBC (8.3%), and PCPG (9.0%) had very low CNV frequency of FGFR3 (Figure 6A). Next, we analyzed the correlation between FGFR3 CNVS and its mRNA expression. As shown in Supplementary Figure 2B, there was no significate correlation was found between FGFR3 CNVs and its mRNA expression across different cancer types (*r* = 0.0127, *p* = 0.2052), suggesting that some other genetic alterations may lead to FGFR3 expression. Mesothelioma (MESO) and DLBC had a relatively high proportion of shallow deletion and were also the tumor type with relatively lower FGFR3 expression. However, KICH and ACC who harbored a relatively high proportion of gain showed a lower expression of FGFR3. Similarly, TGCT, HNSC, CESC, and LUSC had a higher proportion of shallow deletion but was the cancer types with relatively higher expression of FGFR3 (Figure 6A, Supplementary Figure 2A). Furthermore, as shown in Figure 6B, among the 234 samples with FGFR3 mutations described above, 81 samples had FGFR3 CNVs at the same time, of which 32 samples had shallow deletions, 26 samples had gains, 22 samples had amplifications, and one sample had deep deletion. BLCA harbored the highest number of amplification and gain across different cancer types. LUSC and BLCA had the same number and also the highest number of shallow deletions (Figure 6B, Supplementary Table 2).

### Combined FGFR3 alterations (CNVs and mutation) across cancer types

**Figure 7:**
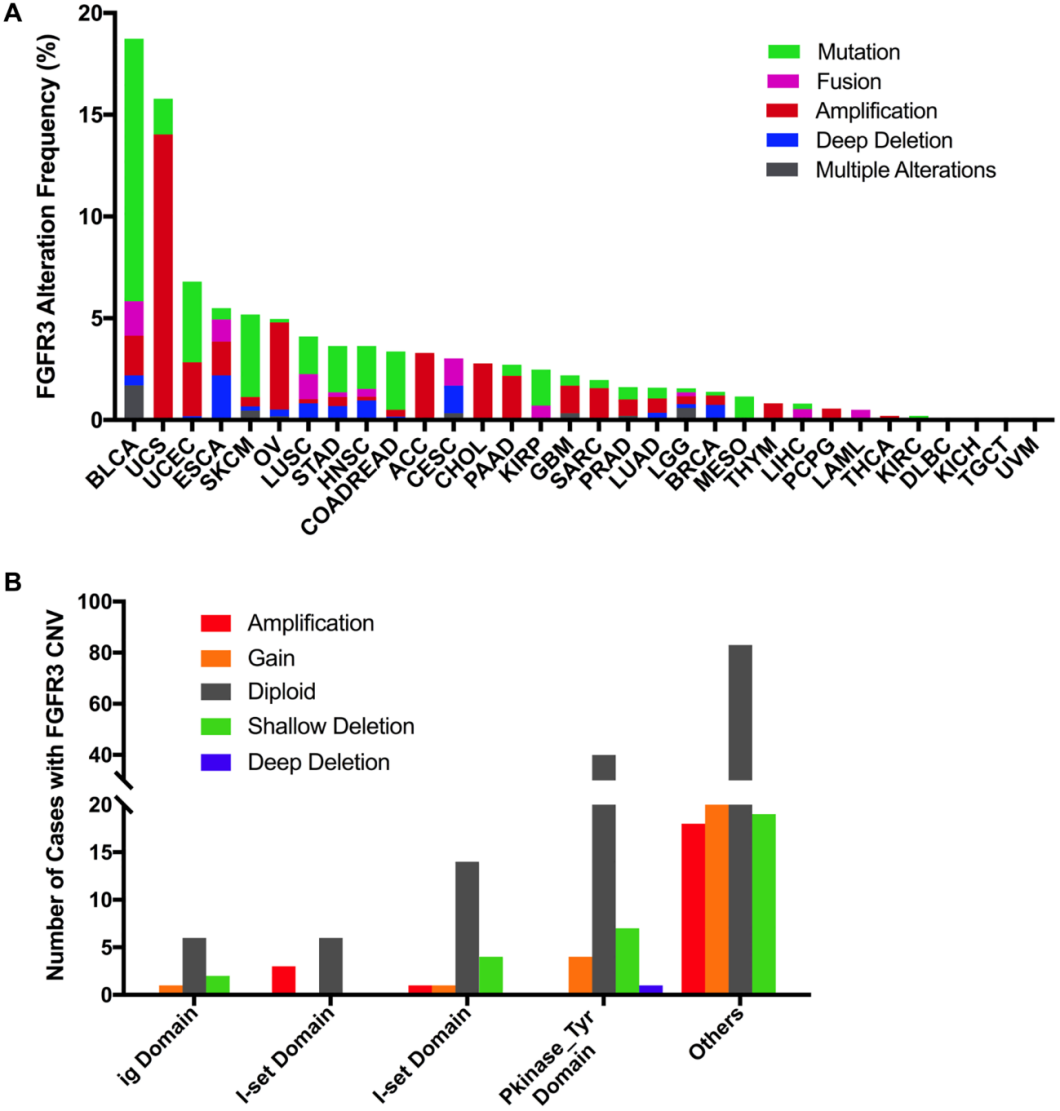
**Pan-cancer analysis of FGFR3 alterations and distribution.** (**A**) The alteration (mutation and CNVs) frequency of FGFR3 across various tumor types. (**B**) The distribution of CNV cases along with mutations located in protein functional domains.

Here, we further analyzed the combined alterations of FGFR3 including mutation and CNVs across different cancer types. The overall alteration frequency of FGFR3 was about 3.2% (detected in 351 of 10,967 samples). As shown in Figure 7A, FGFR3 alterations across different cancer types were quite different. BLCA (18.73%) harbored the most frequency of FGFR3 alterations in which mutation took up a major portion. Other cancers that had dominant FGFR3 mutations but were at relative lower alteration frequency contained UCEC (6.81%), SKCM (5.18%), STAD (3.64%), HNSC (3.63%), COADREAD (3.37%), KIRP (2.47%) and MESO (1.38%). UCS (15.79%) had the second most frequency of FGFR3 alterations with dominant FGFR3 amplification. Similar alteration pattern which was dominant amplification but relatively few mutation was observed in some cancer types such as OV, ACC, CHOL, pancreatic adenocarcinoma (PAAD), GBM, SARC (4.3 vs 0.2%, 3.3 vs 0.0%, 2.8 vs 0.0%, 2.2 vs 0.5%, 1.4 vs 0.5%, 1.6 vs 0.4%, respectively). Fusion was more common in BLCA (1.7%), CESC (1.35%), LUSC (1.23%), and ESCA (1.1%). Deep deletion was mainly distributed in ESCA (2.2%), CESC (1.35%), HNSC (0.96%), LUSC (0.82%), and BRCA (0.74%). Some tumors harbored neither mutations nor CNVs of FGFR3 such as DLBC, KICH, TGCT, and UVM.

Next, we found that mutation location and CNV occurrence of FGFR3 were correlated. As we mentioned above, 234 FGFR3 mutations across 32 TCGA cancers were detected in this analysis. Interestingly, we further found that nearly two-fifths of FGFR3 mutations in the other function-unknown domain were accompanied by shallow deletion, gain, and amplification (57 of 140 mutations). Approximately one-third of FGFR3 mutations in the ig domain, I-set domain (166–245 aa), or I-set domain (260–356 aa) harbored shallow deletion, gain, and amplification (3 of 9 mutations, 3 of 9 mutations, 6 of 20 mutations, respectively). About one-fifth of FGFR3 mutations in the Pkinase_Tyr domain were accompanied by deep deletion, shallow deletion, gain, and amplification (12 of 52 mutations) (Figure 7B).

### FGFR3 alterations and patient survival in different cancer types

**Figure 8:**
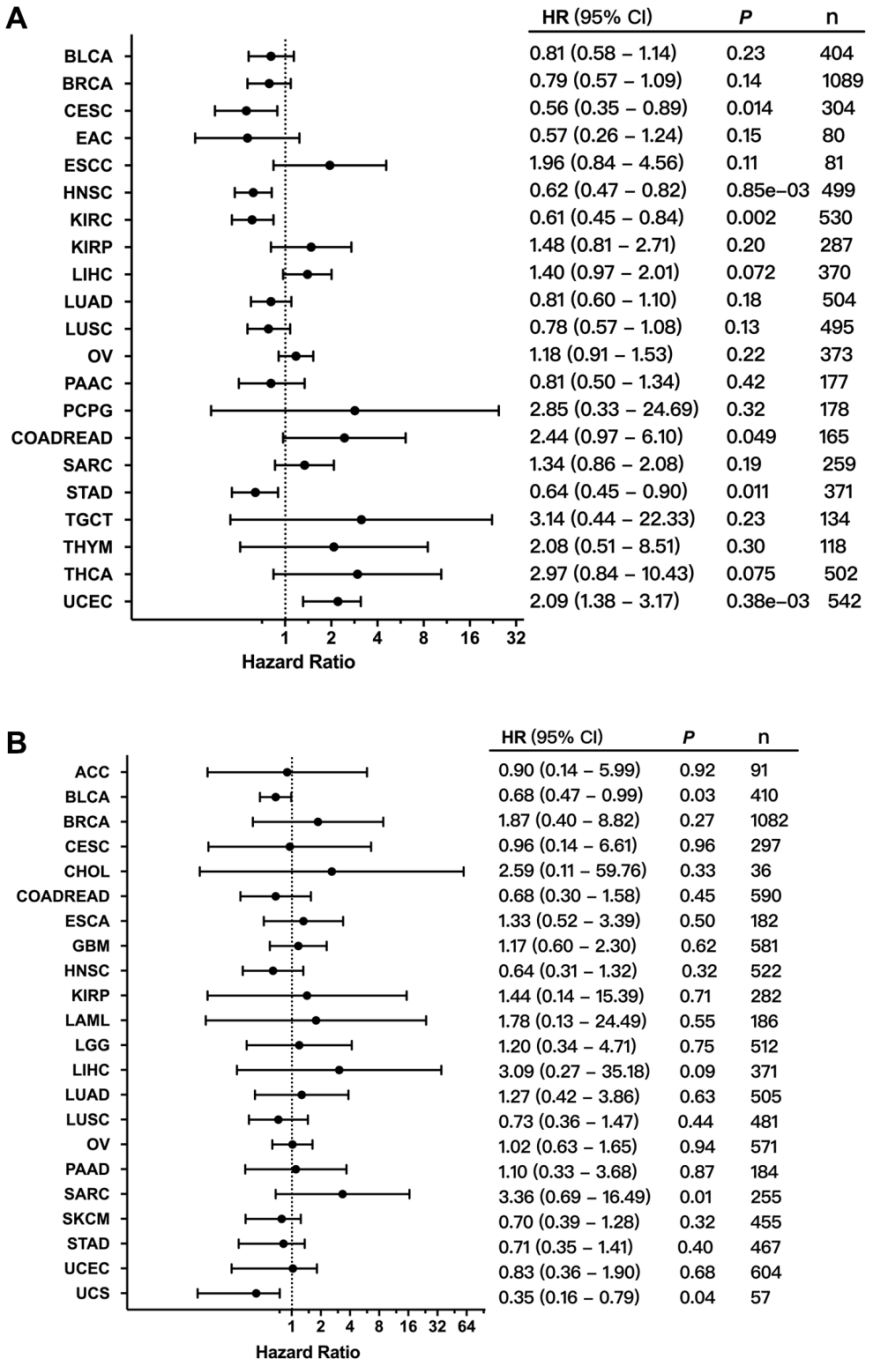
**Correlation between the expression and alterations of FGFR3 and patient survival.** (**A**) The correlation between FGFR3 expression and overall survival (OS) as exhibited in forest plot based on Kaplan-Meier Plotter. (**B**) The correlation between FGFR3 alterations and OS as exhibited in forest plot based on cBioPortal.

In order to evaluate the clinical value of FGFR3 expression, the correlation between the mRNA expression of FGFR3 and patient overall survival (OS) and recurrence-free survival (RFS) was analyzed across different cancer types. As shown in Figure 8A, increased mRNA expression of FGFR3 was correlated with poor patient OS in COADREAD and UCEC. However, the survival results for patients with CESC, HNSC, KIRC, and STAD showed that high mRNA expression of FGFR3 was correlated with better OS. In addition, survival correlation analysis between mRNA expression of FGFR3 and patient RFS across different tumors exhibited that increased mRNA expression of FGFR3 was correlated with poor RFS in ESCC, KIRP, and UCEC, while high mRNA expression of FGFR3 was correlated with better RFS in HNSC, LUSC, and THCA (Supplementary Figure 5). Meanwhile, we further conducted the survival association analysis regarding alteration status across different cancers to explore the clinical value of the FGFR3 alterations. As shown in Figure 8B, FGFR3 alterations were correlated with poor prognosis in SARC, while FGFR3 alterations were correlated with better survival in UCS and BLCA. These opposite survival results may be on account of insufficient sample sizes and different genetic backgrounds.

## DISCUSSION

In this study, the characteristics of FGFR3 across 32 TCGA cancer types were profiled, which were of critical therapeutic and clinical significance. The analysis results showed that FGFR3 expression, methylation, and alteration varied greatly from cancer to cancer. BLCA and UCS had the most frequency of FGFR3 alterations among different cancers. In BLCA, the most common alteration type was the mutation, which was mainly located in the other function-unknown domain, then in the I-set domain. S249C was the most common mutation position in BLCA, followed by the other three mutation sites including Y373C, G370C, R248C. These four mutation sites were all FDA-recognized biomarker predictive of response to an FDA-approved drug, such as erdafitinib [27, 28]. Fusion was also very common in BLCA, FGFR3-TACC3 was the most common fusion type. Patients with metastatic urothelial tumors carrying FGFR3-TACC3 fusion could be also treated with the pan-FGFR targeted inhibitor, erdafitinib [28]. In UCS, amplification was the most common alteration type but mutation was relatively less. The survival association analysis showed that for patients with UCS, FGFR3 alterations were associated with longer overall survival time. In several cancer types amplification accounted for a major proportion of FGFR3 alterations such as OV, ACC, CHOL, PAAD, GBM, and SARC. OV and CHOL harbored high expression of FGFR3, and GBM harbored low expression of FGFR3, however, there was no survival correlation was found between FGFR3 expression and prognosis in these tumors. Some cancer types had dominant mutations but rare CNVs of FGFR3 such as SKCM, COADREAD, KIRP, and MESO. In SKCM, COADREAD, and MESO, most mutations belonged to unknown categories and level NA, and more efforts were needed to figure out their function thus contribute to the clinical treatment. While in KIRP, most mutations were oncogenic and likely oncogenic and belonged to level 3B which was classified by the clinical targeted therapy application potential of FGFR3 somatic mutations. Moreover, ESCA and CESC had the most frequency of deep deletion of FGFR3, but these two cancer types both had high FGFR3 expression, suggesting that some other genetic features may affect the expression of FGFR3. Furthermore, the relatively high frequency of FGFR3 fusion was mainly distributed in BLCA, CESC, LUSC, and ESCA. Several fusion types were detected such as FGFR3-TACC3 and TACC3-FGFR3, TACC3 was the most common partner gene of FGFR3.

The therapeutic landscape of BLCA has dramatically changed in recent years: standard therapy remains platinum chemotherapy, followed by immune checkpoint inhibitors as second-line or maintenance [38–40]. Nowadays, the emergence of FGFR-targeted inhibitors such as erdafitinib was shifting the treatment paradigm for patients with BLCA harboring FGFR3 genetic alterations [16]. In this analysis, we observed that BLCA had the highest frequency of FGFR3 alterations, and mutation took up a major portion. The most common activating point mutations of FGFR3 observed in BLCA from our analysis were S249C, followed by R248C, Y373C, G370C, which were located in exons 7, 15, 10, and 10, respectively. All these four FGFR3 point mutations were oncogenic and belonged to level 1 which was categorized by the clinical targeted therapy application potential of FGFR3 mutations. In addition to these activating FGFR3 point mutations, gene fusion involving FGFR3 such as FGFR3-TACC3 was also commonly detected in BLCA. Patients with BLCA harboring these FGFR3 hotspot mutations or FGFR3-TACC3 fusion were suitable for targeted treatment with an FDA-approved drug, erdafitinib [16]. Erdafitinib, a tyrosine kinase inhibitor (TKI) of FGFR, was approved by the USA FDA in April 2019 for advanced urothelial carcinoma with actionable FGFR2/FGFR3 alterations as the first molecularly targeted therapy [14, 15]. Then the therascreen FGFR RT-PCR kit developed by Qiagen was also approved by FDA as a companion diagnostic test, which was marking a new era of biomarker-driven drug discovery for BLCA. Moreover, erdafitinib was also being explored as a therapy for other FGFR alteration-harboring cancers such as ESCA, CHOL, LIHC, PRAD, and LUSC [16, 41]. In addition to erdafitinib which was for therapy of BLCA with FGFR genetic alterations, other FGFR inhibitors including infigratinib (BGJ398), pemigatinib, rogaratinib, and debio 1347 have been explored under different clinical trials in recent years, exhibiting encouraging clinical data results for BLCA targeted treatment [33, 42–44]. Furthermore, in this analysis, several less common point mutations such as K650E, S371C, and G380R were also observed in BLCA. There was promising clinical evidence that supported these mutation positions as being predictive of response to pan-FGFR-targeted inhibitors such as Debio1347 and BGJ398 [29, 32, 33].

Previous studies reported that FGFR3 genetic fusion was most common in glioma, followed by BLCA. FGFR3 fusion with TACC3 was the classic fusion type that happened in glioma [18]. In this analysis, we found that GBM and LGG had similar mutation rates, and FGFR3 fusion accounted for a major portion. The FGFR3 fusion types observed in glioma (GBM and LGG) in our analysis included FGFR3-TACC3, TACC3-FGFR3, FGFR3-AMBRA1, FGFR3-ELAVL3, FGFR3-FBXO28, and TACC3 was observed to be the most common partner gene of FGFR3 fusion. The first two FGFR3 fusions with TACC3 were oncogenic, and the last three FGFR3 fusion types were likely oncogenic. All of these FGFR3 fusions belonged to level 3B. Patients with glioma harbored FGFR3 fusions were reported to have responded well to FGFR inhibition, and these FGFR3 fusions have been proposed as novel therapeutic targets in glioma [45, 46]. In addition to glioma, FGFR3-TACC3 was also a commonly occurring fusion type in some other cancer types such as BLCA and LUSC. Especially in BLCA, as described above, patients harboring FGFR3-TACC3 could be treated with an FDA-approved drug, erdafitinib [16]. Recent findings revealed that FGFR3-TACC3 could activate oxidative phosphorylation and mitochondrial biogenesis, and engaged oncogenic circuit [47]. More efforts were needed to explore the application of different FGFR inhibitors across various cancers harboring FGFR alterations.

LUAD and LUSC were the two histological types of lung carcinoma [48–50]. Activation of the FGFR family through fusion with various partners has been observed in several cancers, including lung carcinoma. Emerging clinical data showed that these fusions conveyed sensitivity to FGFR inhibitors [51–53]. In our study, we found that LUSC had relatively higher frequency of FGFR3 alterations. FGFR3 fusion was detected in LUSC but not in LUAD, and FGFR3-TACC3 was found to be the most common fusion type. This fusion type was first described in GBM, then subsequently had been observed in various cancer types [54, 55]. TACC3 contained a dimerization domain that could lead to autophosphorylation and activation of FGFR3 signaling [56]. This fusion has been a therapeutic target of an FDA-approved drug, erdafitinib in BLCA, but its application in lung cancer still needs more clinical trials to explore. In addition to fusion, an S249C point mutation was also commonly detected in LUSC but not in LUAD. As described above, this mutation site was the FDA-recognized biomarker predictive of response to a pan-FGFR targeted inhibitor, erdafitinib in BLCA, but the clinical utility of targeted drugs in lung cancer with S249C mutation was still unknown.

Although FGFR3 alterations profile has been reported in several cancer types in previous studies [57], analysis results from these published data could be biased because of additional management during the publication processes. In this report, we profiled FGFR3 expression, methylation, alteration, and their prognostic and clinical implications across 32 TCGA cancer types which were mainly analyzed by the cBioPortal, this approach could unify the TCGA data from different cancer types by adopting ideally processed curation and unified clinical elements [58]. However, there were still some limitations that needed to be mentioned here. First of all, this study was a pan-cancer analysis of FGFR3 genetic alterations and lacked an in-depth investigation and analysis of individual cancer types. Moreover, the sample sizes of some tumor types from this study were not sufficient, and the full FGFR3 alteration spectrum was difficult to achieve in these cancer types. In addition, compared with the alteration frequency of other genes, like BRAF and EGFR, which had an alteration frequency of 8% and 7%, respectively, the frequency of FGFR3 genetic alterations (3%) across different cancer types was not that high which made our evaluation and analysis more challenging and difficult. Several key clues indicated in this study could provide the potential guidance and direction for future investigation.

## CONCLUSIONS

In this study, we first reported the comprehensive pan-cancer profile of FGFR3 genetic alterations and their prognostic and clinical implications across various cancer types of TCGA which was covering over ten thousand tumor samples. Several FGFR3 alterations were more participated in the genesis and development of tumors, while other FGFR3 alterations more participated in targeted treatment. Some tumors with relatively low frequency of FGFR3 genetic alterations were correlated with patient prognosis, while other tumors with a relative high frequency of FGFR3 alterations were not. In conclusion, these analysis results provided a critical novel understanding of FGFR3 deregulation in tumor biology and identified potential therapeutic targets and prognostic indicators for some cancer types.

## MATERIALS AND METHODS

### Data acquisition and reanalysis using different bioinformatics tools

The Genotype-Tissue Expression (GTEx) database is an interactive web resource that collects transcriptome data of widely various tissue types from healthy individuals [59], allowing us to analyze FGFR3 expression in normal tissues. The transcription levels of FGFR3 across different cancer types were extracted from cBioPortal [58, 60], and these transcription data were generated from normalized values with NormalizeExpressionLevels_allsampleref.py which represents the reference population of all samples independent of sample diploid status. All these transcription data of FGFR3 were log10 transformed finally. Then we explored the mRNA expression difference of FGFR3 between tumor tissues and their paired normal tissues across different cancer types or specific cancer subtypes of the TCGA project by using the “Gene_DE” module of TIMER2 (tumor immune estimation resource, version 2) approach. The log2 [TPM (Transcripts per million) +1] transformed expression data were applied for the box plots here. TIMER2 is a bioinformatics platform for systematical analysis of immune infiltrates across various tumor types [61]. For certain cancer types without normal tissues, we further used the GEPIA2 (Gene Expression Profiling Interactive Analysis, version 2) portal to investigate the FGFR3 mRNA expression difference between these tumors and corresponding normal tissues of the GTEx database. Moreover, the GEPIA2 portal also allowed us to further identify violin plots of the FGFR3 expression across different pathological stages of all TCGA cancer types. The log2 [TPM +1] transformed expression data were applied for the violin plots here. GEPIA2 is an interactive web server that analyzes RNA sequencing data from the GTEx and TCGA projects and allows users to conduct the differential expression analyses between tumor and normal tissues, the analysis of patient survival, as well as the access to the profiling of cancer type and pathologic stage, and so on [62]. Furthermore, the GSCALite platform is a comprehensive web server that provides various analysis types including drug sensitivity for genes analysis, genomic variations and their survival analysis, methylation analysis, and so on [63]. We used this platform to explore differential methylation of FGFR3 and downstream genes between tumor tissues and the adjacent normal tissues in different cancer types, and also the association between methylation and the expression of FGFR3 and downstream genes across various cancer types.

Kaplan-Meier Plotter is an online database that enables users to investigate patient survival across various cancer types of TCGA based on large sample datasets [64]. Herein, we used this plotter to analyze the correlation between FGFR3 mRNA expression and patient survival. Next, we downloaded the clinical data from cBioPortal to further identify the correlation between FGFR3 alteration and patient survival across different cancer types. We obtained the hazard ratio (HR), p-values, and the 95% confidence interval (CI), then drew the forest plots to summarize these survival analyses.

cBioPortal is an open comprehensive platform that contains large-scale tumor genomics data and allows users to download and analyze multidimensional tumor genomics and clinical data [58]. In this study, we chose the “TCGA Pan Can Atlas Studies”, and entered “FGFR3” for queries of the genetic alteration characteristics of FGFR3 across various cancer types. This pan-cancer study covered 10,953 patients and 10,967 samples across 32 TCGA cancer types (Supplementary Table 1). For the FGFR3 CNV data, the log ratio value represents: –2 = deep deletion; –1 = shallow deletion; 0 = diploid; 1 = gain; 2 = amplification. For the FGFR3 alteration data, a sample is defined as altered or unaltered (controls) for each gene according to the Onco Query Language (OQL) utilized in the query [37, 58].

### Statistical analyses

The statistical analyses were analyzed with SPSS 12.0 software (IBM Analytics, United States). The statistic calculations on the Mutual Exclusivity tab are performed using all tumor samples from cBioPortal. Student’s *t*-test, linear regression analysis, and Cox regression analysis were conducted when appropriate. *P* < 0.05 was defined as statistically significant if there was no special annotation. The main bioinformatics tools used in this study could be found in Supplementary Table 4.

### Ethical statement

The authors are accountable for all aspects of the work in ensuring that questions related to the accuracy or integrity of any part of the work are appropriately investigated and resolved. None of the data have been previously published or appeared in copyrighted form elsewhere, and not previously published or unpublished data were cited in this paper. No ethics approval was required for this bioinformatics article, as it did not involve patients or patient data.

## Supplementary Materials

Supplementary Figures

Supplementary Table 1

Supplementary Table 2

Supplementary Tables 3 and 4
