# Supplementary material for: Insights of fibroblast growth factor receptor 3 aberrations in pan-cancer and their roles in potential clinical treatment
Source: Aging (Albany NY). 2021 Jun 23;13(12):16541–66. doi: 10.18632/aging.203175 (PMC8266346; doi:10.18632/aging.203175)
Supplement: Supplementary Figures [file aging-13-203175-s001.pdf]

SUPPLEMENTARY FIGURES

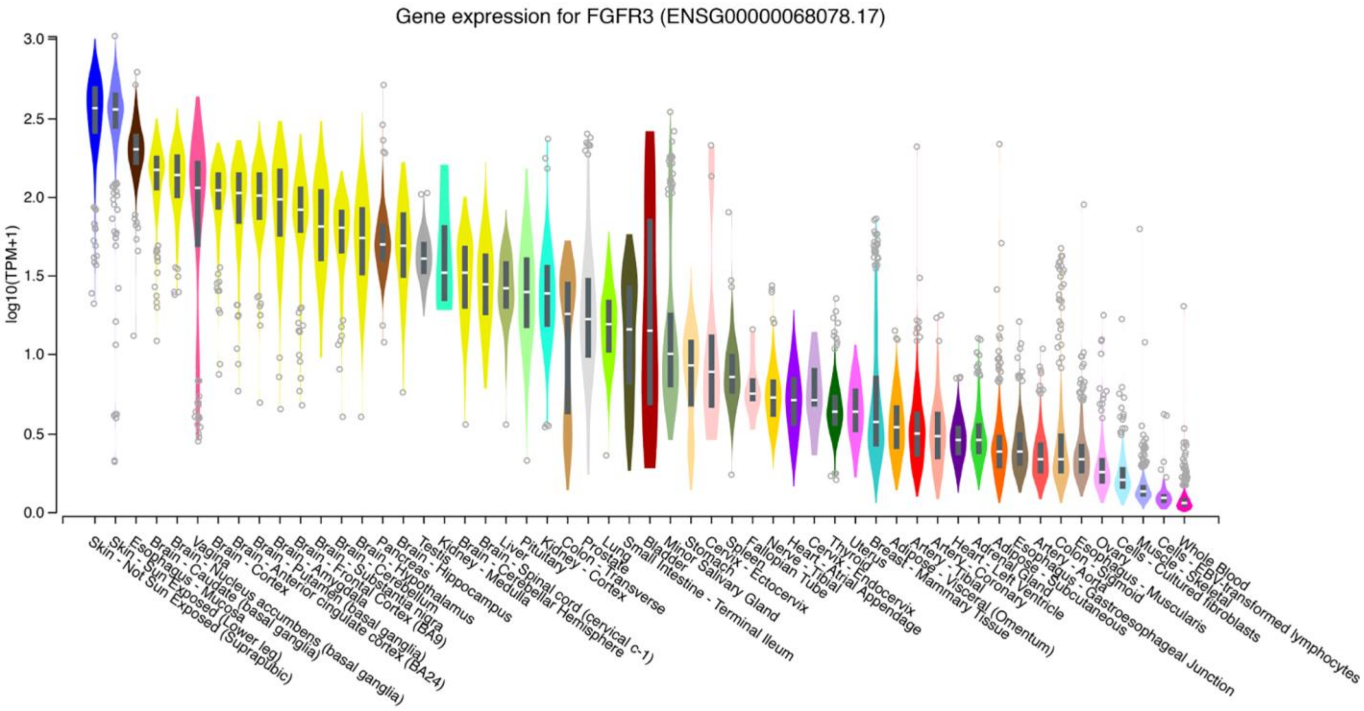

Supplementary Figure 1. FGFR3 expression among 53 types of normal tissues.

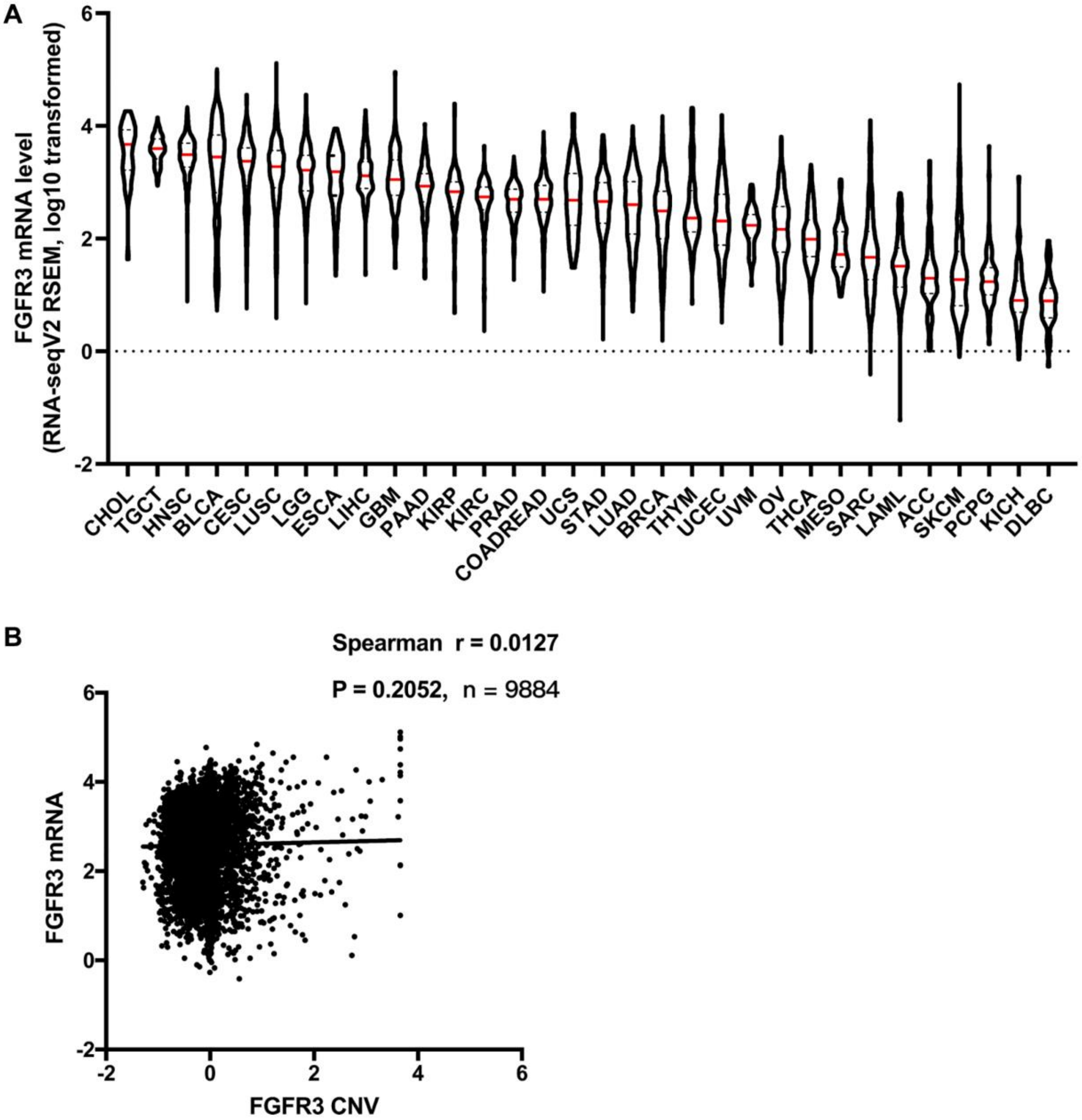

Supplementary Figure 2. FGFR3 mRNA expression across different cancer types. (**A**) FGFR3 mRNA expression across all TCGA tumor types. RNA-seqV2 RSEM and log10 transformed were applied for log-scale. (**B**) The association between mRNA expression and linear CNV of FGFR3 across different tumors.

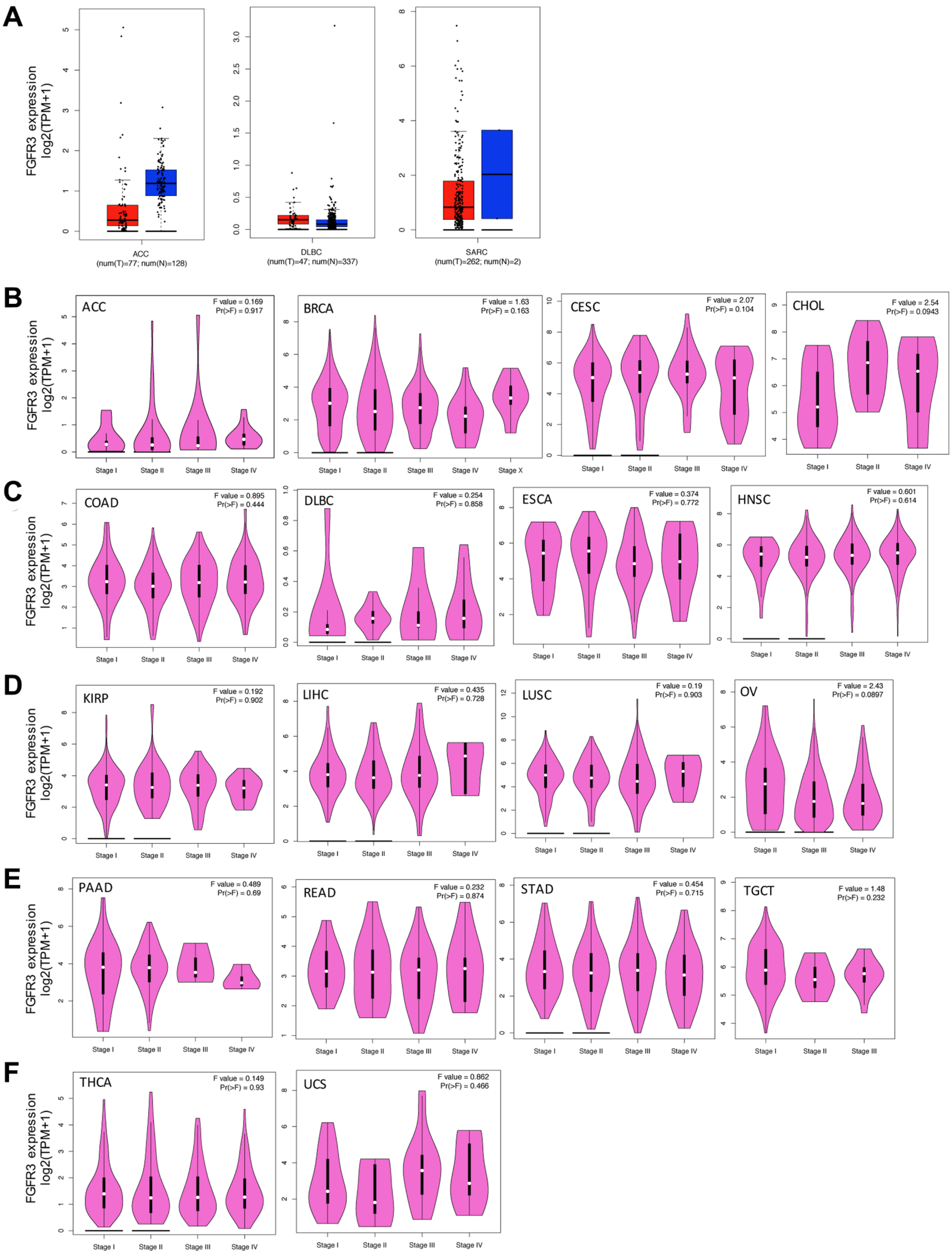

Supplementary Figure 3. FGFR3 mRNA expression in different cancer types and pathological stages. (**A**) The mRNA expression of FGFR3 in ACC, DLBC, and SARC in TCGA project were compared with the corresponding normal tissues of the GTEx database according to GEPIA2. (**B**–**F**) FGFR3 mRNA expression levels were analyzed by the main pathological stages of ACC, BRCA, CESC, CHOL, COAD, DLBC, ESCA, HNSC, KIRP, LIHC, LUSC, OV, PAAD, READ, STAD, TGCT, THCA, and UCS based on GEPIA2. The log2 (TPM +1) was applied for log-scale.

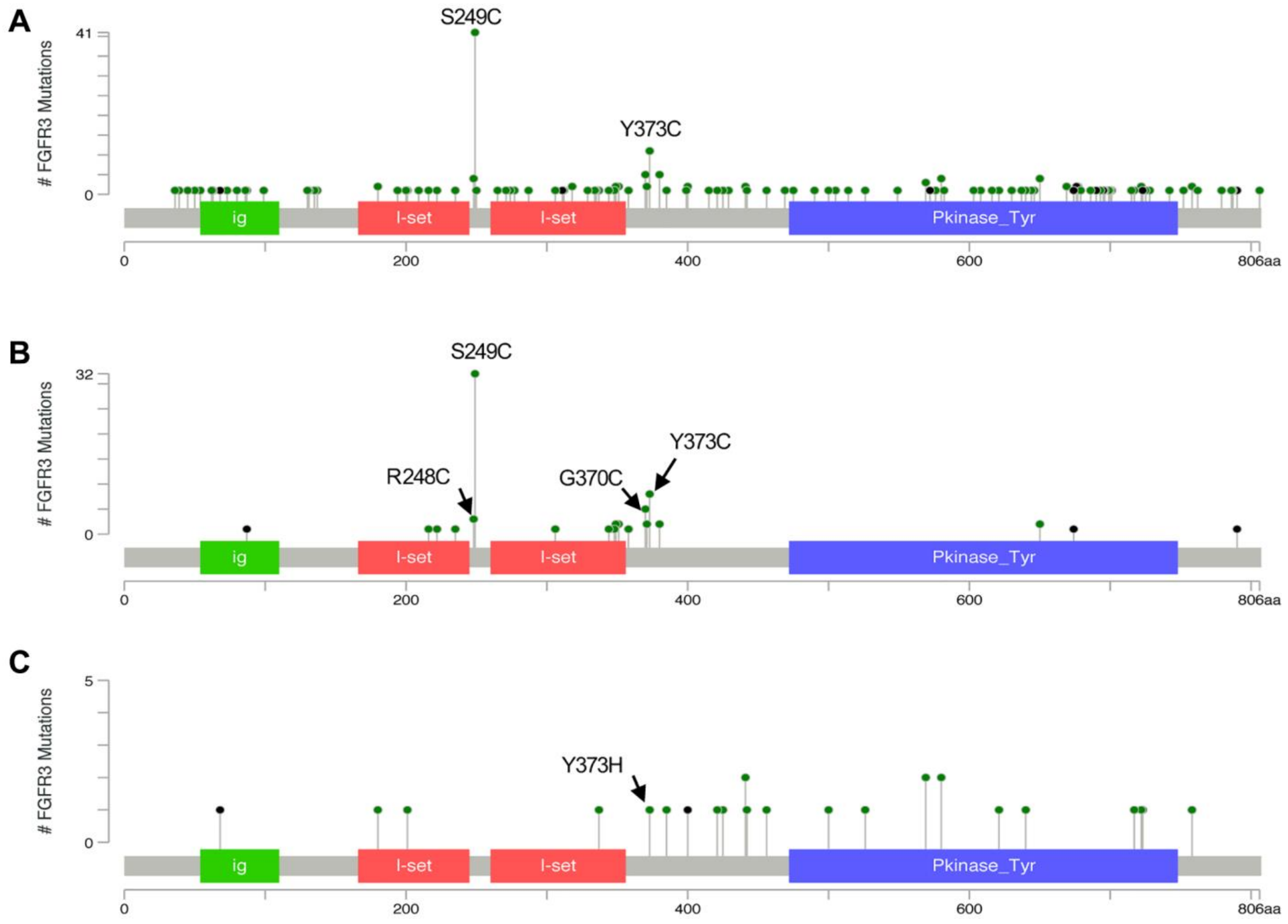

Supplementary Figure 4. FGFR3 mutation distribution in different functional domains. (**A**) FGFR3 mutation distribution in different FGFR3 functional domains in all TCGA tumor types. (**B**) FGFR3 mutation distribution in different FGFR3 functional domains in BLCA. (**C**) FGFR3 mutation distribution in different FGFR3 functional domains in UCEC.

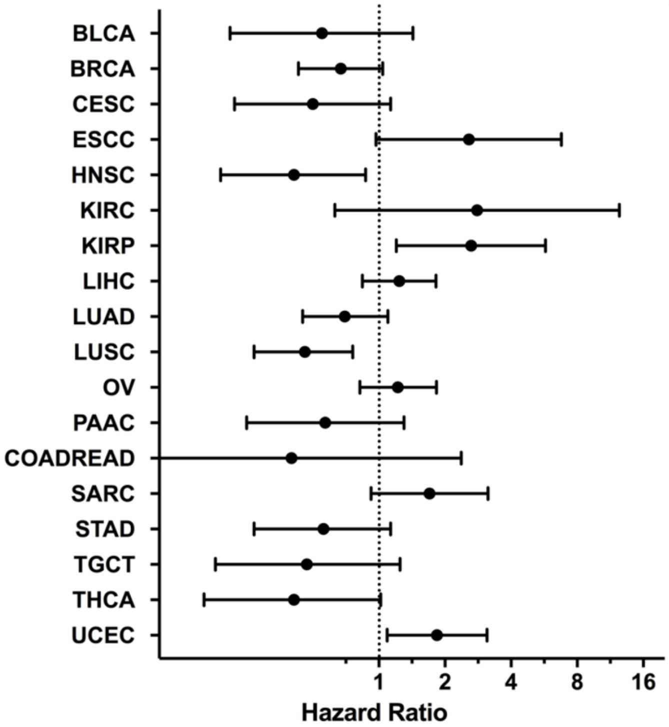

HR (95% CI)	*P*	n
0.55 (0.21 – 1.43)	0.21	187
0.67 (0.43 – 1.04)	0.072	947
0.50 (0.22 – 1.13)	0.088	174
2.57 (0.97 – 6.77)	0.049	54
0.41 (0.19 – 0.87)	0.017	124
2.80 (0.63 – 12.46)	0.16	117
2.63 (1.20 – 5.74)	0.012	183
1.24 (0.84 – 1.82)	0.29	316
0.70 (0.45 – 1.10)	0.12	330
0.46 (0.27 – 0.76)	0.0019	300
1.22 (0.82 – 1.83)	0.33	177
0.57 (0.25 – 1.30)	0.18	67
0.40 (0.07 – 2.37)	0.30	47
1.70 (0.92 – 3.14)	0.089	152
0.56 (0.27 – 1.13)	0.10	215
0.47 (0.18 – 1.25)	0.12	105
0.41 (0.16 – 1.02)	0.047	353
1.84 (1.09 – 3.11)	0.022	422

Supplementary Figure 5. The correlation between FGFR3 expression and recurrence-free survival (RFS) as exhibited in forest plot based on Kaplan-Meier plotter.
