# Supplementary material for: Insights of fibroblast growth factor receptor 3 aberrations in pan-cancer and their roles in potential clinical treatment
Source: Aging (Albany NY). 2021 Jun 23;13(12):16541–66. doi: 10.18632/aging.203175 (PMC8266346; doi:10.18632/aging.203175)
Supplement: Supplementary Table 1 [file aging-13-203175-s002.pdf]

SUPPLEMENTARY TABLE

Supplementary Table 1. Summary of 32 TCGA tumor types and sample size.

TCGA Cancer Abbreviation	TCGA Cancer Type	Sample Number
ACC	Adrenocortical carcinoma	92
BLCA	Bladder urothelial carcinoma	411
BRCA	Breast invasive carcinoma	1084
CESC	Cervical squamous cell carcinoma and endocervical adenocarcinoma	297
CHOL	Cholangiocarcinoma	36
COADREAD	Colon adenocarcinoma/Rectum adenocarcinoma	594
DLBC	Lymphoid neoplasm diffuse large B-cell lymphoma	48
ESCA	Esophageal carcinoma	182
GBM	Glioblastoma multiforme	592
HNSC	Head and Neck squamous cell carcinoma	523
KICH	Kidney chromophobe	65
KIRC	Kidney renal clear cell carcinoma	512
KIRP	Kidney renal papillary cell carcinoma	283
LAML	Acute myeloid leukemia	200
LGG	Brain lower grade glioma	513
LIHC	Liver hepatocellular carcinoma	372
LUAD	Lung adenocarcinoma	566
LUSC	Lung squamous cell carcinoma	487
MESO	Mesothelioma	87
OV	Ovarian serous cystadenocarcinoma	585
PAAD	Pancreatic adenocarcinoma	184
PCPG	Pheochromocytoma and Paraganglioma	178
PRAD	Prostate adenocarcinoma	494
SARC	Sarcoma	255
SKCM	Skin cutaneous melanoma	448
STAD	Stomach adenocarcinoma	440
TGCT	Testicular germ cell tumors	149
THCA	Thyroid carcinoma	500
THYM	Thymoma	123
UCEC	Uterine corpus endometrial carcinoma	529
UCS	Uterine carcinosarcoma	57
UVM	Uveal Melanoma	80
