# Supplementary material for: Insights of fibroblast growth factor receptor 3 aberrations in pan-cancer and their roles in potential clinical treatment
Source: Aging (Albany NY). 2021 Jun 23;13(12):16541–66. doi: 10.18632/aging.203175 (PMC8266346; doi:10.18632/aging.203175)
Supplement: Supplementary Tables 3 and 4 [file aging-13-203175-s004.pdf]

SUPPLEMENTARY TABLES

Supplementary Table 3. FGFR3 somatic mutation distribution in different protein functional domains for tumors with FGFR3 mutations and all tumors together.

	ig: Immunoglobulin domain (54-110aa)	I-set: Immunoglobulin I-set domain (166-245aa)	I-set: Immunoglobulin I-set domain (260-356aa)	Pkinase_Tyr: Protein tyrosine kinase (472-748aa)	Others	Fusion
Pan-cancer	9	9	20	54	100	42
BLCA	1	3	7	3	54	8
UCEC	1	2	1	11	9	
SKCM	5		2	10	4	1
COADREAD	1	3	1	8	5	
LUSC			1	1	7	7
HNSC			1	1	11	2
STAD	1		3	4	2	1
KIRP				4	3	2
LGG				1	1	5
CESC						6
GBM				3		3
PRAD			1	2		1
ESCA				1		2
LIHC		1				2
LUAD			1	1	1	
BRCA			1	1		
OV				1		1
PAAD			1	1		
KIRC				1		
LAML						1
MESO					1	
SARC					1	
UCS					1	

Supplementary Table 4. The main bioinformatics tools used to analyze the role of FGFR3 in pan-cancer.

Database	Samples	URL	References
cBioPortal	Tissues	http://cbioportal.org/	[58]
GTEx	Tissues	http://www.gtexportal.org/home/	[59]
TIMER2	Tissues	http://timer.cistrome.org/	[61]
GEPIA2	Tissues	http://gepia.cancer-pku.cn/	[62]
GSCALite	Tissues	http://bioinfo.life.hust.edu.cn/web/GSCALite/	[63]
Kaplan-Meier Plotter	Tissues	http://kmplot.com/	[64]
